# Cardiopulmonary Exercise Test: Background, Applicability and Interpretation

**DOI:** 10.5935/abc.20160171

**Published:** 2016-11

**Authors:** Artur Haddad Herdy, Luiz Eduardo Fonteles Ritt, Ricardo Stein, Claudio Gil Soares de Araújo, Mauricio Milani, Romeu Sérgio Meneghelo, Almir Sérgio Ferraz, Carlos Hossri, Antonio Eduardo Monteiro de Almeida, Miguel Morita Fernandes-Silva, Salvador Manoel Serra

**Affiliations:** 1 Instituto de Cardiologia de Santa Catarina, São José, SC; 2 Universidade do Sul de Santa Catarina (UNISUL), Florianópolis, SC; 3 Sociedade Brasileira de Cardiologia (SBC), Rio de Janeiro, RJ; 4 Hospital Cardio Pulmonar da Bahia, Salvador, BA; 5 Hospital Santa Izabel da Santa Casa de Misericórdia da Bahia, Salvador, BA; 6 Serviço de Cardiologia - Universidade Federal do Rio Grande do Sul, Porto Alegre, RS; 7 Grupo de Pesquisa em Cardiologia do Exercício do Hospital de Clínicas de Porto Alegre, Porto Alegre, RS; 8 Vitta Centro de Bem Estar Físico, Porto Alegre, RS; 9 Instituto do Coração Edson Saad Universidade Federal do Rio de Janeiro, Rio de Janeiro, RJ; 10 Clínica de Medicina do Exercício, CLINIMEX, Rio de Janeiro, RJ; 11 Clínica Fitcordis Medicina do Exercício, Brasília, DF; 12 Instituto Dante Pazzanese de Cardiologia, São Paulo, SP; 13 Hospital Israelita Albert Einstein, São Paulo, SP; 14 Hospital do Coração (HCOR), São Paulo, SP; 15 Hospital Universitário Lauro Wanderley e Departamento de Educação Física (UFPB), João Pessoa, PB; 16 Cardio Lógica Métodos Diagnósticos, João Pessoa, PB; 17 Brigham and Women's Hospital, Harvard Medical School, Boston, MA - USA; 18 Instituto Estadual de Cardiologia Aloysio de Castro (IECAC), Rio de Janeiro, RJ - Brazil

**Keywords:** Exercise Test, Exercise, Evaluation, Lung Volume Measurements, Oxygen Consumption

## Abstract

Cardiopulmonary exercise test (CPET) has been gaining importance as a method of functional assessment in Brazil and worldwide. In its most frequent applications, CPET consists in applying a gradually increasing intensity exercise until exhaustion or until the appearance of limiting symptoms and/or signs. The following parameters are measured: ventilation; oxygen consumption (VO_2_); carbon dioxide production (VCO_2_); and the other variables of conventional exercise testing. In addition, in specific situations, pulse oximetry and flow-volume loops during and after exertion are measured. The CPET provides joint data analysis that allows complete assessment of the cardiovascular, respiratory, muscular and metabolic systems during exertion, being considered gold standard for cardiorespiratory functional assessment.^1-6^

The CPET allows defining mechanisms related to low functional capacity that can cause symptoms, such as dyspnea, and correlate them with changes in the cardiovascular, pulmonary and skeletal muscle systems. Furthermore, it can be used to provide the prognostic assessment of patients with heart or lung diseases, and in the preoperative period, in addition to aiding in a more careful exercise prescription to healthy subjects, athletes and patients with heart or lung diseases.

Similarly to CPET clinical use, its research also increases, with the publication of several scientific contributions from Brazilian researchers in high-impact journals.

Therefore, this study aimed at providing a comprehensive review on the applicability of CPET to different clinical situations, in addition to serving as a practical guide for the interpretation of that test.

## Major variables and their meanings

**Oxygen consumption (VO_2_):** is the volume of O_2_ extracted from the air inhaled during pulmonary ventilation in a period of time. It is usually expressed in mL.min^-1^ or L.min^-1^ (STPD). In practice, maximum VO_2_ (VO_2_ max) is defined as the highest value reached, despite progressive increase of the load applied, with the development of a plateau in the VO_2_ curve during an incremental exercise test. When no plateau can be identified, the highest value obtained at the end of an exhausting exercise is characterized as peak VO_2_, which, in practice, is used as VO_2_ max. Mean values at intervals of 10 to 60 seconds should be measured depending on the protocol (short-interval means for protocols with short stages and longer-interval means for protocols with longer stages). The response is influenced by a central mechanism (cardiovascular and/or pulmonary) and peripheral function (skeletal muscle).^1-6^ The normal values depend on several factors, such as: age, sex, weight, height, physical activity level, genetic variability and ethnicity. Different equations to predict the normal values of VO_2_ max or peak VO_2_ have been determined from different populations. Although the equation proposed by Wasserman and Whipp^6^ is the most frequently used, a national equation^7^ seems to be more suitable for Brazilians.

The term 'peak VO_2_' is used as a synonym for VO_2_ max throughout this text. Peak VO_2_ is considered abnormal when below 85% of the predicted value.^6^ It has been used as a universal marker^1-3,5^ that can broadly reflect disease severity in patients with heart failure (HF), pulmonary hypertension, hypertrophic cardiomyopathy (HCM), chronic obstructive pulmonary disease (COPD) and restrictive pulmonary disease, in addition to physical fitness level.^1-5,8^ The VO_2_ value measured in the first ventilatory threshold (VT1) or anaerobic threshold (AT) is determined by the nonlinear increase of pulmonary ventilation (VE) in relation to VO_2_. From the physiological viewpoint, AT represents the upper limit of workloads during exercise, which can be sustained over a prolonged period of time without progressively increasing blood lactate and consequent pulmonary hyperventilation.^6^ Peak VO_2_ and AT values are influenced by genetic predisposition, diseases, exercise and aerobic training types. The normal mean AT values expected for adults are around 40% to 65% of peak VO_2_.^6^ The AT values are important for the individualized prescription of exercise, as well as for the diagnosis of anemia, physical unfitness, myopathies and cardiopathies in the presence of values lower than the predicted ones.^2-6^

**Pulmonary ventilation (VE):** expressed as liters per minute, is the volume of air moved in and out of the lungs. It is determined as the product of respiratory rate by the volume of air exhaled at every cycle (tidal volume). At rest, 7 to 9 L/min are ventilated, but in athletes that value can reach 200 L/min at maximal exertion.^6^ Ventilation increases continuously during progressive effort on CPET and undergoes additional increases influenced by the anaerobic metabolism resulting from the accumulation of lactic acid, well defined as the first and second ventilatory thresholds. Periodic (or oscillatory) ventilation is defined as the resting oscillatory pattern that persists in ≥ 60% of the effort with an amplitude ≥ 15% as compared to mean resting values.^9^ It reflects disease severity and relates to worse prognosis in patients with HF.^3-5^

**Respiratory coefficient or respiratory exchange ratio (R):** expresses the ratio between CO_2_ production and O_2_ consumption (VCO_2_/VO_2_). It is currently the best non-invasive indicator of maximal or quasi-maximal exercise intensity. Values above 1.0 can reflect intense exercise, but those ≥ 1.10 are those searched on CPET, and have been accepted as a parameter of exhaustion or quasi-exhaustion.^3,7^

**Ventilatory equivalents for oxygen (VE/VO_2_) and for carbon dioxide (VE/VCO_2_):** are the ratios between pulmonary ventilation and O_2_ consumption (VE/VO_2_) or CO_2_ production (VE/VCO_2_). Both decline from rest to submaximal exercise intensities, with VE/VO_2_ reaching minimum values before AT, when its progressive increase occurs, caused by the increase in ventilation to eliminate extra CO_2_ production. That results in lactate buffering by blood bicarbonate.^6^ Later, VE/VCO_2_ increases (respiratory compensation point - RCP, or second ventilatory threshold - VT2), resulting from ventilatory increase (compensatory respiratory alkalosis) in response to blood pH reduction due to the progressive accumulation of lactic acid at muscle level.^6^ The VE/VO_2_ reflects the ventilatory need for a certain O_2_ consumption level, being, thus, an index of ventilatory efficiency. Patients with inadequate ratio between pulmonary ventilation and pulmonary perfusion (increased physiological dead space) ventilate inefficiently and have high VE/VO_2_ values (pulmonary disease and HF).^6^ Peak values above 50 have been useful to diagnose patients suspected of having mitochondrial myopathy.^10^ On the other hand, VE/VCO_2_ represents the ventilatory need to eliminate a certain amount of CO_2_ produced by active tissues, being influenced by partial pressure of carbon dioxide (PaCO_2_). In addition, VE/VCO_2_ slope is the relationship between VE, plotted in the Y axis, and VCO_2_, in the X axis, both measured as L/min. The VE/VCO_2_ slope can be determined in submaximal tests.^11^ It relates to changes in the ventilation-perfusion relationship or hyperventilation. The VE/VCO_2_ slope reflects the severity and prognosis of patients with HF, pulmonary hypertension, HCM, COPD and restrictive pulmonary disease.^1,3-5,8,11,12^

**End-tidal CO_2_ partial pressure (PETCO_2_):** reflects ventilation-perfusion within the pulmonary system, and, indirectly, cardiac function.^6^ Its value ranges from 36 to 42 mmHg, with 3- to 8-mmHg elevations during moderate intensity exercise, reaching a maximal value with subsequent drop, due to VE increase, characterizing RCP.^1^ Abnormal values can represent disease severity in patients with HF, HCM, pulmonary hypertension, COPD and restrictive pulmonary disease.^3-6,8,12^

**Oxygen pulse (O_2_ pulse):** is the ratio between VO_2_ (mLO_2_/min) and heart rate (HR - bpm). Its meaning is better understood by observing the Fick equation: VO_2_ = HR x systolic volume (SV) x arteriovenous oxygen difference [(A-V)O_2_ diff]. Considering that, in many clinical situations, (A-V)O_2_ diff does not substantially change in incremental exercise, O_2_ pulse represents SV, and, in a way, left ventricular performance. Thus, VO_2_ ≅ HR x SV or VO_2_/HR ≅ SV. Under certain circumstances, the morphological analysis of its curve aids in the diagnosis of ventricular dysfunction and important effort-induced myocardial ischemia.^1,3-6^

**Breathing reserve (VE/MVV):** represents the ratio between maximal ventilation during exercise (VE) and maximum voluntary ventilation (MVV) at rest, both variables in L/min. Equations to predict MVV can be used (forced expiratory volume in the first second - FEV_1_ x 40), although it can be measured directly on pre-test spirometry. Normal values are greater than 0.20. However, in both athletes and those performing strenuous exercises, a higher fraction of breathing reserve can be physiologically used. It is useful in the differential diagnosis of dyspnea related to pulmonary mechanism.^6^

**ΔVO_2_/ΔWR Relationship:** relationship between VO_2_ (Y axis in mL.min^-1^) and workload (X axis in Watts), measured only during exercise on a cycle ergometer with ramp protocol, whose value is progressively and linearly incremented until maximal effort. It is useful in the diagnosis of patients suspected of having myocardial ischemia with left ventricular dysfunction on exertion. Its normal value for adults is 9 mL.min^-1^.W^-1^ (the lowest limit being 8.6 mL.min^-1^.W^-1^).

Other variables: the minimum VE/VO_2_ value is the cardiorespiratory optimal point (COP).^13^ It is a submaximal variable that reflects the best integration between the respiratory and cardiovascular systems. Although it is easy to obtain, further studies are required to determine its clinical applicability and prognostic meaning. Oxygen uptake efficiency slope (OUES) was widely studied, being measured by the relationship between VO_2_ and the logarithmic transformation (base 10) of VE. The OUES provides information on the severity of HF.^14^ Similarly to VE/VCO_2_ slope, it does not require a maximal test. T_1/2_VO_2_ is the time necessary for a 50% drop in VO_2_ measured at peak exercise (from the beginning of recovery) until the third minute of recovery. It decreases with physical training and its increase is negatively associated with the prognosis of HF patients.^15^ Circulatory power is the product of peak systolic blood pressure (SBP) by peak VO_2_, while ventilatory power is peak SBP divided by VE/VCO_2_ slope. Both have prognostic value in HF.^16^ Finally, the association of CPET with measurements of cardiac output and SV, by use of non-invasive hemodynamic analysis (impedance cardiography - ICG), can provide variables, such as Δ*Q*/ΔVO_2_ slope to assess coronary artery disease (CAD), HF and some myopathies.^10^

### Functional assessment and CPET-based aerobic exercise prescription

The CPET is considered the best method to assess aerobic performance, and, mainly, to support aerobic exercise prescription.^17,18^ Considered class IIa indication - optimized prescription of exercise to healthy individuals, individuals with heart or lung diseases entering a program of regular exercise - and class IIb indication - athletes -,^1^ it is still rarely used with such purposes by clinical cardiologists.

By use of the joint analysis of exhaled gases, work and/or exertion performed and the behavior of hemodynamic variables, mainly HR, a more comprehensive functional assessment can be obtained. Thus, a more precise and individualized program of aerobic exercise can be outlined. Apparently healthy individuals who engage in moderate- to high-intensity aerobic practice can benefit from CPET regarding exercise prescription and performance assessment.^18^ For individuals with heart diseases and high-performance athletes, such benefits have been widely established. Prescription errors, both insufficiency and excess, in such individuals can have a negative impact on the results expected from a training program.

Briefly, for the prescription of aerobic exercises, the most relevant data obtained from CPET are HR and exercise intensity at which the ventilatory thresholds occur, especially, AT or VT1.^19^ The exercise intensity at which VT1 occurs characterizes the highest submaximal level tolerated by a certain individual for long time periods. Because that exercise intensity varies even between two individuals with identical maximal functional capacity (and even with similar maximal VO_2_ values measured), its precise determination via CPET enhances and refines the quality of aerobic exercise prescription. In practical terms, HR values in different points of maximal CPET are used to establish the bases for a more objective prescription. More often, the following values are considered: HR at rest with the individual lying down (resting HR), maximal HR (HRmax), HR at AT, HR at RCP, and HR at the 'R = 1' point. Traditionally, exercises have been prescribed based on the intensity related to HR, but the workload related to thresholds and maximal effort can also be used.^1,20^ When the objective is to train up to a moderate subjective intensity that can be sustained for long periods, we set the limit at the AT. Between the AT and RCP, the exercise intensity is higher, but usually still tolerated for prolonged periods, with wide individual variations. Finally, the training can be performed above the RCP, with very intense and much more difficult to sustain exercises, which can be of the interval type (alternating resting periods with some type of mild-to-moderate intensity exercise).^20^

There are numerous protocols that can be used for both healthy individuals and those with diverse patologies.^21^ These protocols are used to prescribe steady-state aerobic exercise (walking or running) or interval exercise, with an important component of "anaerobic" exercise, alternating rhythms and intensities (alternate walking and running, up and down walking and cycling, ball sports and spinning classes).

However, the quality of that prescription, based on HR derived from CPET, depends on some factors. It is convenient that CPET be performed with a ramp protocol, minimum duration of eight minutes, on an ergometer more similar to the aerobic exercise that will be prescribed (cycle ergometer for cyclers, treadmill for runners). Longer protocols tend to allow greater differentiation and precision in identifying the exercise intensity that corresponds to the thresholds. It is worth noting that data collected during a CPET performed in an air-conditioned room can differ from those obtained during a walk or cycling or even a long running (more than 45 minutes) at open air locations and under more adverse climate conditions, in which there may be a cardiovascular drift^22^ phenomenon, characterized by a progressive increase of HR, instead of remaining in steady-state, despite of a constant intensity of exercise. However, for patients using HR-controlling devices or on regular use of medications with negative chronotropic action, specific care should be taken so that the HR-based prescription obtained on CPET can remain valid. The most obvious case is that of patients on beta-blockers on a single daily dose, which make HR during exercise vary according to the time interval between medication administration and exercise performance.^23^ To minimize that chronopharmacological effect, such patients should undergo CPET at the time closest to that of regular exercise. In patients with pacemakers, resynchronization devices and atrial fibrillation, the HR measured by these HR sensors is inaccurate. For those individuals and some athletes whose training intensity is based on load or velocity, exercise can be prescribed based on velocities or loads relative to thresholds. Some studies have suggested that the load relative to 'R = 1' bears the best correlation with maximal exertion in metabolic balance.^24^

Finally, other potentially relevant variables can be obtained via exhaled breath analysis, including some that do not require maximal exertion, such as mechanical efficiency analysis and COP,^13^ which widens the CPET value for prescription of primarily aerobic exercises. 

### CPET in heart failure

Chronic heart failure (CHF) is a systemic syndrome, and reduced functional capacity is one of its main features. The cardiovascular deficit has a direct influence on other organs and systems, such as the pulmonary, renal and skeletal muscular ones. CPET is considered "gold standard" for the functional assessment of patients with CHF, propitiating diagnostic and prognostic data derived from direct measurement of VO_2_, VCO_2_ and VE. In addition, the variables VE/VO_2_, VE/CO_2_, VCO_2_/VO_2_ and R, as well as the metabolic points AT and RCT, are useful parameters to indicate accurately the maximal aerobic capacity, to quantify functional restriction, to measure the response to drug therapy and to guide physical training prescription.

The Brazilian Society of Cardiology guidelines for the management of patients with CHF present CPET as class I indication in the assessment of both heart transplantation candidates and dyspnea mechanisms. The use of CPET is class II indication for exercise prescription, and to assess the severity, prognosis and responses to therapeutic interventions in CHF.^25,26^

The response to CPET of a patient with CHF is characterized by: reduced VO_2_, AT < 40% of the predicted VO_2_ max, O_2_ pulse < 85% and as a plateau, increased VE/VCO_2_, reduced OUES, wide breathing reserve and usually normal O_2_ saturation.^2^ Peak VO_2_ is the specific and direct measure of functional capacity. Several studies have shown its independent prognostic capacity in CHF. According to the Brazilian guidelines for heart transplantation, a peak VO_2_ lower than 10 mL.kg^-1^.min^-1^ is class I indication for that procedure, while a peak VO_2_ below 12 mL.kg^-1^.min^-1^ (patients on beta-blocker) or below 14 mL.kg^-1^.min^-1^, is class IIa indication, particularly for those with other criteria of worse prognosis (VE/VCO_2_ slope > 35).^27^ Weber et al.^28^ have proposed a classification for peak VO_2_ results: class A = VO_2_ > 20 mL.kg^-1^.min^-1^; class B = VO_2_ 16-20 mL.kg^-1^.min^-1^; class C = VO_2_ 10-15 mL.kg^-1^.min^-1^; and class D = VO_2_ < 10 mL.kg^-1^.min^-1^. It is worth noting that, for peak VO_2_ value to have prognostic accuracy, the test has to meet the requirements of a maximal test (proposed for HF: R > 1.05, at least).

Other important variables measured via CPET that add independent prognostic value for patients with CHF are: VE/VCO_2_ slope, OUES, T_1/2_VO_2_, HR recovery in the first post-exertion minute, presence of periodic ventilation, and PETCO_2_ and O_2_ pulse behaviors.

Chua et al.,^29^ assessing patients with CHF using CPET, have observed those with VE/VCO_2_ slope > 34 were at higher risk for hospitalization due to decompensation, and for death. Other authors,^30-32^ assessing the prognostic value of VE/VCO_2_ slope in CHF, have shown it to be a variable with excellent independent value, even higher than that of peak VO_2_, and important to patients who reach only submaximal exertion. In a population with CHF due to Chagas disease, Ritt et al.^33^ have reported that the best cutoff point for worse prognosis was VE/VCO_2_ slope > 32.5, thus earlier than those reported by studies on other etiologies. Arena et al.^34^ have published the following ventilatory classes based on VE/VCO_2_ slope values: class I, VE/VCO_2_ ≤ 29.9; class II, 30-35.9; class III, 36-44.9; class IV, ≥ 45. In 2 years, event-free survivals (death, transplantation or implantation of ventricular assistance device) for classes I-IV were 97.2%, 85.2%, 72.3% and 44.2%, respectively (P < 0.0001). Assessing a population of patients via CPET for heart transplantation, Ferreira et al.^35^ have found a VE/CO_2_ slope cutoff point of ≥ 43 as ideal to determine the indication for heart transplantation. The use of VE/VCO_2_ slope as a criterion for selection of candidates for transplantation could reclassify correctly 18.3% more patients than the classic peak-VO_2_-based criteria (p < 0.001).^35^

In addition, OUES has an independent prognostic value. Initially, Baba et al.^36^ have described that variable behavior, whose cutoff point and independent prognostic value were subsequently assessed by other authors. A cutoff point < 1.47 L/min determines a group with more severe CHF.^37,14^

T_1/2_VO_2_ is identified in patients with CHF. Studying patients with VO_2_ ≥ 15, between 10.1 and 14.9, and ≤ 10 mL.kg^-1^.min^-1^, Groote et al.^38^ have reported T_1/2_VO_2_ values of 108 ± 44.6, 137 ± 58.7, and 176 ± 75 seconds, respectively.^38^ In patients with no heart disease, T_1/2_VO_2_ is usually < 90 seconds.^39^

The kinetics of HR recovery (HRR) is a well-established prognostic marker in patients with CAD,^40^ related to changes in post-exertion autonomic balance. In CHF, it is also an independent factor of mortality, even in patients on beta-blockers.^41^ The cutoff point established for that population was ≤ 16 bpm in an active recovery protocol (hazard ratio: 4.6; 95%CI: 2.8-7.5; p < 0.001). Its clinical usefulness has been assessed for heart transplantation indication in patients in the intermediary zone of peak VO_2_ (VO_2_ 10.1-13.9 mL.kg^-1^.min^-1^), in whom, the HRR analysis aggregated value to peak VO_2_ and VE/VCO_2_ slope. The prognosis of patients with altered HRR and VE/VCO_2_ slope was comparable to that of those with VO_2_ < 10 mL.kg^-1^.min^-1^.^42^

Wide oscillations in ventilation during exertion relates to cardiovascular events and death in patients with CHF. That pattern, analogous to the Cheyne-Stokes respiration, was named periodic ventilation. The occurrence of periodic ventilation during exertion (characterized by an amplitude variation > 5 L/min for at least three cycles) was related to an up to three-fold higher mortality in patients with CHF (hazard ratio: 2.97; 95%CI: 1.34 - 6.54; p < 0.007).^9,43,44^ The presence of periodic ventilation increased the risk of patients with reduced peak VO_2_ and elevated VE/VCO_2_ slope.^45^

Another index that reflects the dynamics of pulmonary changes and CO_2_ diffusion at alveolar level is PETCO_2_ at rest. Mean values < 33 mmHg after 2 minutes at rest were independently correlated with worse prognosis and greater mortality in CHF (hazard ratio: 2.17; 95%CI: 1.48-3.19; p < 0.001).^46^

O_2_ pulse can be assessed regarding its absolute value and its behavior during exertion. A plateau is usually related to an insufficient increase in SV on exertion. An O_2_ pulse < 85% of the predicted value correlates independently with major cardiovascular events in CHF. Among patients with peak VO_2_ < 14.3 mL.kg^-1^.min^-1^ and O_2_ pulse < 85% of the predicted values, mortality was greater than among those with only one of those parameters altered (hazard ratio: 4.76 versus 2.31, respectively). O_2_ pulse could also reclassify the risk of patients into intermediate zone of peak VO_2_ for transplantation (10-14 mL.kg^-1^.min^-1^). Patients in the O_2_ pulse < 85% zone had mortality similar to those with VO_2_ < 10 mL.kg^-1^.min^-1^.^47^

Each CPET variable correlates with the interaction of HF with another organ or system. Thus, the joint analysis of those variables can better stratify the risk of those patients. The CPET variables can be combined into risk scores in CHF. Levy et al. have shown that the addition of VE/VCO_2_ slope data to Seattle Heart Failure Model could improve the prognostic ability of that score, reclassifying 40% of the patients into a more appropriate risk category (p = 0.002).^48^

To determine the prognostic significance of CPET in CHF, Cahalin et al. have conducted a meta-analysis of studies published until 2013 and calculated the odds ratios (OR) of each prognostic variable. The OR of the main prognostic variables assessed (peak VO_2_, VE/VCO_2_ slope, OUES and periodic ventilation) were 4.10 (CI: 3.16-5.33), 5.40 (CI: 4.17-6.99), 8.08 (CI: 4.19-15.58) and 5.48 (CI: 3.82-7.86), respectively.^49^

For those not dealing with CPET on a daily basis, the assessment of each variable can be unpractical. Myers et al.^50^ have developed a score that combines the information of the main CPET variables into a number. The points are attributed as follows: VE/VCO_2_ slope ≥ 34 - 7 points; HRR ≤ 16 bpm - 5 points; OUES ≤ 1.4 - 3 points; PETCO_2_ < 33 mmHg - 3 points; peak VO_2_ ≤ 14 mL.kg^−1^.min^−1^ - 2 points. The score ranges from 0 to 20, 0-5 being the reference. The others correlated in an increasing manner with the risk of death/transplantation or implantation of ventricular assistance device: 6-10 (hazard ratio: 2.74, 95%CI: 2.16-3.48; p < 0.001), 11-15 (hazard ratio: 4.6, 95%CI: 3.55-5.98; p < 0.001) and > 15 (hazard ratio: 9.25, 95%CI: 5.75-14.88; p < 0.001). In three years, the mortality of patients with score >15 was 12.2%, in comparison to 1.2% in those with score < 5. A recent analysis^51^ applied that score to class B patients according to Weber heart failure classification (analogous to NYHA class II). In the three-year follow-up, patients with score ≥ 10 had an event-free survival equivalent to that of Weber class C patients, and those with score < 10 had a prognosis equivalent to that of Weber class A patients.

**Figure 1:**
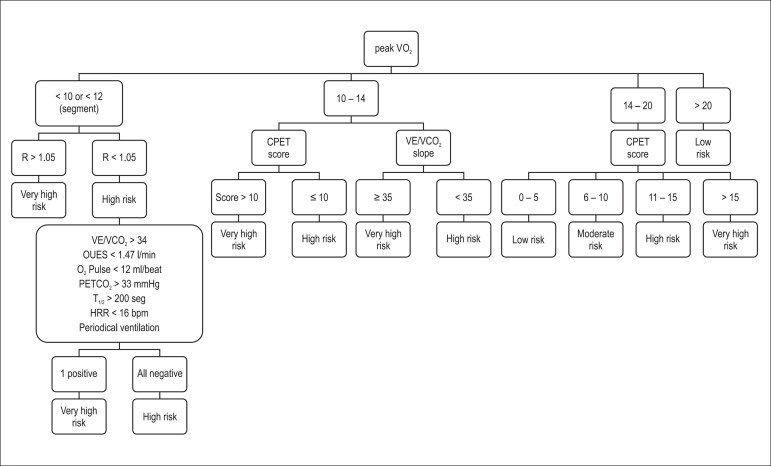
Risk stratification based on CPET results from patients with CHF (Modified from Ribeiro JP, Stein R, Chiappa GR. J Cardiopulm Rehabil. 2006 Mar- Apr;26(2):63-71). CPET: cardiopulmonary exercise test; VO_2_ : oxygen consumption; R: respiratory exchange ratio; VE/VCO_2_ slope: ratio between pulmonary ventilation and carbon dioxide production; PETCO_2_ : extrapolated end-tidal carbon dioxide tension; T_1/2_ : time necessary for a post-exertion 50% drop in VO_2_ measured; OUES: oxygen uptake efficiency slope; HRR: heart rate recovery.

The CPET plays a preponderant role in the assessment of patients with CHF, not only regarding the selection of candidates for transplantation, but also to determine the prognosis and help with the therapeutic decision. Figure 1 shows a stratification strategy that combines those variables.

### CPET to assess myocardial ischemia

CPET can help to assess myocardial ischemia in patients with suspected CAD, a clinical condition where a significant ischemic load, during exercise, is expected to negatively influence systolic myocardial performance.^1^ During incremental exercise, the myocardial unbalance between O_2_ offer and demand triggers a sequence of metabolic changes that can ultimately lead to the insufficient physiological elevation of SV. On CPET, this is observed as a depressed, in plateau or declining shape curve of O2 pulse.

Three CPET variables are indicated to assess the presence and severity of myocardial ischemia: 1) O_2_ pulse; 2) VO_2_ curve and elevation; and 3) relationship between VO_2_ variation and load variation, in watts, in this case, exclusively, on cycle ergometer.^52^

### Oxygen pulse and oxygen consumption curve

Usually, (A-V)O_2_ diff tends to remain constant during incremental exertion, except for rare cases of anemias, hemoglobinopathies, some congenital heart diseases and COPD, in which there is a significant drop in peripheral oxygen saturation. Except for those clinical conditions, one can infer that SV behavior during incremental exercise is reflected by the equation: VO_2_/HR = SV. The VO_2_/HR ratio, called "oxygen pulse" and measured in milliliters per beat, reflects the O_2_ volume ejected into the aorta at every systole. Likewise, SV, also measured in milliliters per beat, reveals the blood volume ejected into the aorta at every systole. Thus, those two variables, despite being numerically different, reflect left ventricular hemodynamic behavior during CPET.

**Figure 2:**
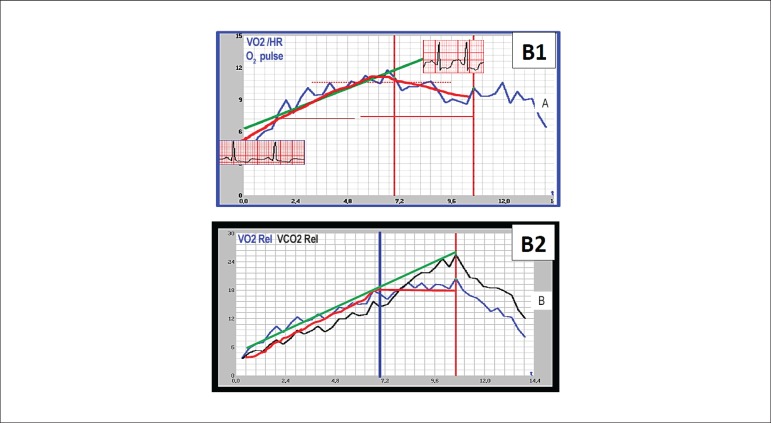
Cardiopulmonary exercise test in the pre-rehabilitation assessment of a 57-year-old hypertensive, diabetic, overweight male patient with three-vessel coronary disease, who refused to undergo myocardial revascularization surgery eight years earlier. A) evident drop in oxygen pulse. B) early plateau of oxygen consumption. Both changes (A and B) were due to ischemic depression of the ST segment (evident in A), followed by progressive chest pain.

The analysis of the VO_2_/HR curve as a function of time, which should have the increasing morphology of a parabola, is as important as the numerical O_2_ pulse value during the incremental phase of CPET. The identification of a curve with a plateau or decline indicates a reduction in O_2_ pulse and SV during exercise, and can indicate myocardial ischemia^53^ (Figure 2). It is worth noting that other clinical conditions can cause similar changes, such as ventricular dysfunctions due to non-ischemic cardiomyopathies, providing prognostic information on HF with reduced ejection fraction,^3^ and obstructive valve heart diseases. In the presence of severe chronotropic changes, artificial electric stimulation and arrhythmias, such as atrial fibrillation, O_2_ pulse analysis becomes compromised and inaccurate.

### ΔVO _2_/ΔWR Ratio (Watts)

**Figure 3:**
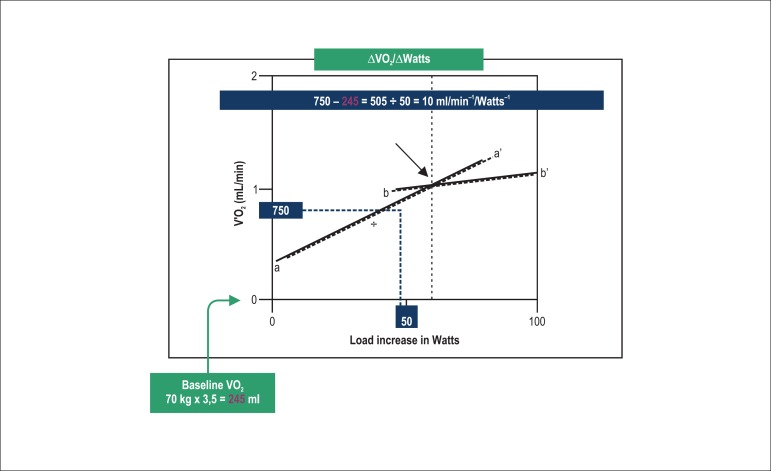
The ΔVO_2_ /ΔWR relationship around 10mL.min.Watts suddenly reduces, despite the exercise intensity increase. This loss of linear relationship could indicate the presence of myocardial ischemia by use of CPET performed on a cycle ergometer (modified from reference 52).

To every increase in load imposed during CPET, a similar increase in VO_2_ is expected. Normally, a 1-Watt increment in workload should correspond to a 10 mL.min^-1^-increase in absolute VO_2_. The loss of this linear relationship, with a reduction of slope often to less than 5 mL.min^-1^.Watt^-1^, despite the increase in exercise intensity during CPET, contributes to the diagnosis of myocardial ischemia^52^ (Figure 3).

It is worth noting that the changes suggestive of ischemia on CPET become more evident as ischemia severity increases. The CPET variables should be analyzed in light of the pre-test clinical suspicion. CPET can be indicated for functional assessment of patients with established CAD, as well as for the investigation of myocardial ischemia diagnosis, mainly in the following conditions:

1. When there is moderate to high pre-test likelihood of myocardial ischemia;
2. To increase diagnostic accuracy of myocardial ischemia, when, on CPET, there is clinical, hemodynamic or electrocardiographic change, hindering the diagnosis via conventional exercise test;
3. In the presence of a large ischemic myocardial area, hindering left ventricular function due to SV reduction during exercise;
4. For follow-up assessment after percutaneous or surgical revascularization;
5. Similarly to other clinical conditions, CPET can be recommended to assess the prognosis of patients with CAD, with or without evidence of ischemia, by using other variables usually used for that purpose, such as VE/VCO_2_ slope, peak VO_2_, OUES, periodic ventilation and T_1/2_VO_2_, in addition to other CPET variables that wait for more solid studies.^6,54-57^

### CPET in the differential diagnosis of dyspnea

Dyspnea is a common symptom in several clinical situations, characterized by the perception of respiratory difficulty or discomfort. Its pathophysiology is complex, involving neuro-humoral and mechanical mechanisms. From the practical viewpoint, the differential diagnosis can be classified into four categories: cardiac, pulmonary, mixed cardiopulmonary, and non-cardiopulmonary.^58,59^

The use of CPET for dyspnea assessment can be divided into two settings - patients with dyspnea without an established diagnosis and patients with multiple possible causes - in whom the test is useful to determine which mechanism prevails and causes symptoms. The dyspnea, whose cause cannot be elucidated via history, physical examination and complementary tests at rest, should be better assessed by using CPET. By use of joint analysis, from rest to maximal exertion, the cardiovascular, respiratory and peripheral metabolism responses can provide information on the dyspnea mechanism. Because of its low cost, CPET can be indicated early in the investigative hierarchy of dyspnea assessment, serving to guide other complementary tests, when required, for therapeutic management and prognostic assessment (Table 1).

**Table 1:** Behavior of major CPET variables in several causes of dyspnea

Dyspnea origin	Cardiovascular	Pulmonary	Vascular-pulmonary	Hyperventilation	Fake
Variables					
VO_2_	reduced	reduced	reduced	normal	reduced
AT	early	normal	early	normal	normal
R	normal	reduced	normal/reduced	normal/reduced	reduced
VE/VCO_2 _slope	high	high	high	high	normal
PETCO_2_	low	low	low at AT	low at AT	normal
VE/MVV	normal	reduced	normal	normal	normal
O_2 _pulse	reduced/plateau	normal/plateau	reduced/plateau	normal	normal
O_2_ Sat	normal	drop	drop	normal	normal
ΔVO_2_/ΔWR	reduced/plateau	normal/plateau	reduced/plateau	normal	normal

VO2: oxygen consumption; AT: anaerobic threshold; R: respiratory exchange ratio; VE/VCO_2_ slope: ratio between pulmonary ventilation and carbon dioxide production; PETCO2: extrapolated end-tidal carbon dioxide tension; VE/MVV: ventilatory reserve; O2 Sat: oxyhemoglobin saturation; ΔVO2/ΔWR: relationship between oxygen consumption and workload.

Studies on the clinical value of CPET in patients with chronic dyspnea (more than 1 month) of undetermined origin or dyspnea of multiple causes have evidenced practical use: to differentiate dyspnea of cardiocirculatory primary origin from dyspnea of pulmonary ventilatory etiology or that related to problems in the ventilation-perfusion binomial; to quantify the different mechanisms of multiple-cause dyspnea; to identify an unsuspected or underestimated circulatory component; and to identify a psychogenic or simulation component.^60,61^

The differential diagnosis of those pathologies requires pragmatic interpretation of CPET data.^62^ The first step is to assess peak VO_2_ and to determine the percentage of the predicted value achieved. Pulmonary, cardiovascular and metabolic diseases or physical unfitness can account for VO_2_ reduction. Then, breathing reserve should be assessed, and, when low, it can identify underlying pulmonary disease. Breathing reserve lower than 20% is found in pulmonary diseases; however, as already described, highly-trained individuals or those in situations of extreme exertion can also consume their ventilatory reserve on maximal exertion as a compensatory mechanism, but, in such cases, peak VO_2_ will not be significantly reduced.

The following step is the analysis of O_2_ saturation. A drop greater than 4% on peak exertion as compared to resting is characteristic of pulmonary limitation. High VE/VCO_2_ slope and PETCO_2_ < 33 mmHg at rest and/or elevation greater than 8 mmHg during exertion suggest respiratory mechanisms as the cause of dyspnea.^3,63^

Observation of O_2_ pulse and ΔVO_2_/ΔWR ratio can identify heart disease, if the curves show plateau or decline, reflecting an inadequate SV to the load imposed.^64^ However, individuals with lung disease and some degree of pulmonary hypertension can also develop a plateau of O_2_ pulse. The combination of plateau of O_2_ pulse with a decrease in O_2_ saturation, VE/VCO_2_ slope > 40 and reduced PETCO_2_ (< 33 mmHg at rest or < 36 mmHg at AT) strongly suggests pulmonary hypertension or a pathology with pulmonary vascular impairment.^3,63,65^

Patients with dyspnea due to cardiovascular limitation have reduced VO_2_, early AT, ventilatory inefficiency (high VE/VCO_2_ slope), inefficient O_2_ uptake (reduced OUES), plateau of O_2_ pulse or of ΔVO_2_/ΔWR ratio, with normal ventilatory reserve, PETCO_2_ < 33 mmHg at rest and/or increase < 3 mmHg during exertion, in addition to lack of drop in O_2_ saturation.^3,63^

Patients with physical unfitness and anemia have reduced VO_2_ and increased ΔVO_2_/ΔWR (cycle ergometer), but they do not meet the criteria for pulmonary or cardiovascular limitation. Extremely physically unfit patients can have reduced AT and increased HR/VO_2_ ratio. On the other hand, a low R, despite the sensation of extreme fatigue on BORG scale, points to a peripheral mechanism as the cause of limitation to exertion.

Patients with hyperventilation have reduced ventilatory efficiency (high VE/VCO_2_ slope), reduced PETCO_2_ at AT, sudden changes in the ventilatory pattern with phases of tachypnea and hypopnea, and extremely increased respiratory rate on exertion. Usually, the ventilatory reserve is normal and O_2_ saturation has a physiological behavior.

Studying 39 patients with asthma of difficult control, McNicholl et al. have reported that, in 14 of them, the persistent complaint of dyspnea was explained by hyperventilation, preventing the undue increase of the dose of bronchodilators in those patients.^66^ In legal situations, facing a complaint of dyspnea, the medical expert can have difficulty to determine if the symptom is true or to establish an effective symptom graduation, and CPET can be used to clarify the scenario. To diagnose fake dyspnea by using CPET: the patient reports extreme fatigue, asks for exertion interruption and shows normal ventilatory reserve, normal O_2_ saturation behavior, AT within the expected range for the maximal VO_2_ predicted (40%-60%), but an R compatible with submaximal exertion (<1), in addition to apparent chronotropic deficit.

## CPET in pulmonary diseases

### Chronic obstructive pulmonary disease

The severity of COPD is determined based on symptoms and spirometry results. Pulmonary function tests at rest, however, do not accurately predict the grade of intolerance to exertion.^64^ The inability to increase ventilation to levels that allow high gas exchange is one of the mechanisms that explain dyspnea on exertion. That phenomenon can be observed on CPET and is usually interpreted as ventilatory limitation. Although characteristic of obstructive scenarios, it can occur in restrictive diseases, such as interstitial pulmonary diseases, and in abnormalities of the thoracic cage. The criterion that defines ventilatory limitation is arguable, but, when the breathing reserve at peak exertion is lower than 15%, limitation is considered to occur, especially when R is lower than 1.0.^67^

**Figure 4:**
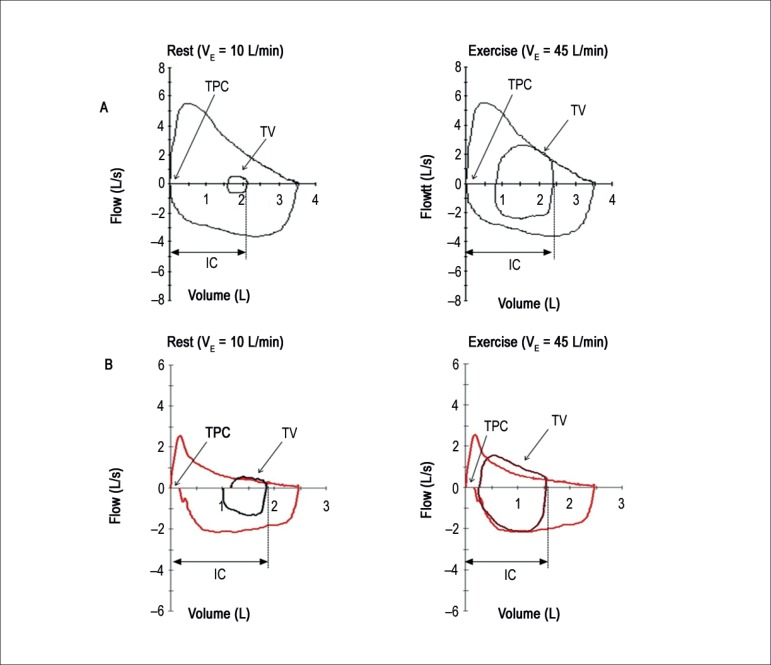
Flow-volume curves: A) patient without pulmonary disease; B) patient with chronic obstructive pulmonary disease. Note loop displacement to the left with overlapping. IC: inspiratory capacity; TPC: total pulmonary capacity, TV: tidal volume.

In patients with COPD, peak VO_2_ continues to be the best index of aerobic capacity, as long as patients exercise to their limit. However, other aspects should be considered when interpreting the CPET of patients with COPD. There is a combination of low ventilatory capacity and high ventilatory demand, increasing the sensation of dyspnea. The perception of lower limb exertion is often exaggerated in such patients and can be a limiting factor, especially in tests performed on a cycle ergometer.^2^ Another factor that can significantly contribute to the development of unbearable dyspnea during exercise is dynamic hyperinflation. With the increase of respiratory flow during exercise, the air is held in the lungs, causing a progressive increase in residual volume, thus reducing the inspiratory capacity (Figure 4). That frequently occurs together with a reduction in tidal volume, indicating that the respiratory mechanics has reached its functional limit. Dynamic hyperinflation can be observed on CPET when periodic analyses of the flow-volume curve occurs with inspiratory capacity measured during exercise. That is especially useful when symptom intensity and the grade of airway obstruction is disproportional.^62^

### Exertion-induced bronchospasm

Exertion-induced bronchospasm (EIB) is the acute narrowing of airways resulting from exercise. Its clinical manifestations include "chest wheezing", cough, dyspnea or chest pressure usually 5 to 10 minutes after exercise, and, less commonly, during exercise. Its diagnosis requires a specific protocol with repeated post-exertion spirometry, typically at 5, 10 and 15 minutes. A drop in FEV1 equal to or greater than 10% as compared to that of pre-exertion is diagnosed as EIB.^67^ For the diagnosis of bronchial hyperreactivity, that test is less sensitive than bronchoprovocation challenge tests with bronchoconstrictors (methacholine, histamine), being, however, more specific for the diagnosis of EIB.

### Early detection of pulmonary vascular disease

In addition, CPET has been used to the early detection of pulmonary vascular disease. However, the pathophysiological aspects of pulmonary hypertension are worth considering to understand and interpret the findings in the clinical context.

Pulmonary hypertension is defined as mean pulmonary artery pressure (mPAP) equal to or greater than 25 mmHg,^68,69^ and dyspnea on exertion is usually its earliest symptom. The pulmonary circulation has high capacitance, and normal mPAP values are frequently observed at the early stages of pulmonary vascular disease. For an increase in mPAP levels at rest to occur, more than 50% of the pulmonary circulation needs to be obstructed, resulting in a relatively late diagnosis of pulmonary vascular disease.^69,70^

The identification of pulmonary hypertension during exertion requires the use of a pulmonary artery catheter for direct measurement during exercise. This is part of the invasive (or advanced) CPET, available only at a few centers. One limitation is that the definition of pulmonary hypertension on exertion, mPAP greater than 30 mmHg, is arbitrary,^69,71^ and healthy individuals can reach much higher values.^72^ In addition, there are not enough data to conclude that patients with that "abnormal hemodynamics" will progress to true pulmonary hypertension at rest.

CPET can provide information to help the clinician suspect pulmonary hypertension when assessing a patient with dyspnea of undefined etiology. The VE/VCO_2_ ratio at AT and peak exertion areSBP lower than 120 mmHg had extremely elevated in patients with pulmonary hypertension, higher than that of patients with HF and same functional class.^73^ In addition, low PETCO^2^ values at the end of expiration, both at rest and exercise, were associated with pulmonary hypertension.

It has been suggested that, in the absence of acute hyperventilation (normal R), VE/VCO_2_ ratio greater than 37 and PETCO^2^ below 30 mmHg at AT could indicate pulmonary vascular disease. Exceptionally low PETCO^2^ values (below 20 mmHg) are uncommon in other diseases and increase the suspicion of pulmonary hypertension in patients assessed for dyspnea on exertion.^74^

### Prognostic assessment in pulmonary hypertension

CPET can be used to assess both the severity of pulmonary hypertension in patients with established disease and the response to therapy. Studying idiopathic pulmonary arterial hypertension, Wensel et al. showed that individuals with peak VO_2_ lower than 10.4 mL.kg-1.min^-1^ and peak SBP lower than 120 mmHg had worse prognosis.^75^ The guidelines of the European Society of Cardiology^69,70^ recommend that peak VO_2_ values greater than 15.0 and lower than 12.0 mL.kg^-1^.min^-1^ indicate good and bad prognosis, respectively. However, that parameter should not be assessed isolated, but be part of a comprehensive assessment to determine pulmonary hypertension severity.

In addition, VE/VCO_2_ ratio at AT and VE/VCO_2_ slope have been associated with pulmonary hypertension prognosis, with values equal to or greater than 54 and 62, respectively, indicating shorter survival.^73^ However, that relationship seems not to apply to all forms of pulmonary hypertension. A more elevated VE/VCO_2_ slope was observed in pulmonary hypertension due to chronic pulmonary thromboembolism as compared to pulmonary arterial hypertension. It is worth noting that, in pulmonary hypertension due to chronic pulmonary thromboembolism, VE/VCO_2_ slope did not associate with functional class, suggesting no relationship with severity and high values at early phases.^76^ Another parameter associated with the worse survival of patients with pulmonary arterial hypertension is the presence, on CPET, of signs of right-to-left shunt during exercise.^77^

Figure 4: Flow-volume curves: A) patient without pulmonary disease; B) patient with chronic obstructive pulmonary disease. Note loop displacement to the left with overlapping.

### CPET in children and adolescents

In the pediatric population, the use of CPET is similar to that of the adult population, but with specific particularities related to the childhood universe. Environmental conditions should allow children to adapt to the test, therefore enabling good performance assessment.^78^

CPET has been very useful to assess healthy individuals and those with complex congenital heart diseases,^79^ allowing the determination of pathophysiological causes that limit functional capacity.^80^ Protocols and ergometers (treadmill and cycle ergometer) are selected according to the objectives and experience of the laboratories conducting the tests. Ramp protocols, however, are currently the most often applied.

Comparing the cardiorespiratory responses of healthy children with those of healthy young adults, Prado et al.^81^ have evidenced lower cardiovascular (evidenced by lower O_2_ pulse) and respiratory (lower PETCO_2_) efficiencies, higher respiratory rate and VE/VO_2_ at peak exercise and at AT level. However, healthy children have higher metabolic efficiency (lower R and peak VO_2_, similar to those of healthy young adults).

The literature indicates possible reasons for the immaturity of the anaerobic metabolism of children during physical exercise, such as lower muscular glycogen levels,^82^ reduced activity of phosphofrutokinase-1 ^83^ and of lactate dehydrogenase,^84^ and higher proportion of muscle fibers of slow contraction.^85^

In addition, children with heart diseases usually have lower aerobic potency than young adults and children without heart diseases.^86^ Other variables derived from CPET are extremely useful to measure the response to exercise. OUES indicates systemic and pulmonary perfusion, and correlates strongly with peak VO_2_. In children without heart diseases, OUES increases with their development.^80^ However, according to the study by Dias et al.,^86^ in congenital heart disease, an association was identified between OUES and functional impairment severity in 59 children in the late postoperative period of congenital heart disease correction. Those authors have reported that reduced OUES was associated with low peak VO_2_ (below 80% of the predicted value) in 90% of the cases, confirming the presence of a cardiovascular disorder during exertion.

In addition, ergospirometric assessment has been extremely useful in the follow-up of partially or completely treated complex congenital heart diseases, as an aid to indicate the ideal time for new therapeutic interventions. Table 1 shows the performance of children in the late postoperative period of several cyanogenic congenital heart diseases, such as Fallot tetralogy, transposition of the great arteries and single ventricle heart.

**Table 2:** * Comparison of the ergospirometric performance of children with complex congenital heart disease and healthy ones undergoing maximal incremental test

	Heart disease (n = 30)	Normal (n = 30)	p
**Age**	11.8 ± 6.2	11.9 ± 6.7	NS
**Incremental test performance**			
Max. velocity (km.h^-1^)	9.8 ± 3.1	10.9 ± 4.9	0.001
AT velocity (km.h^-1^)	5.7 ± 1.7	6.9 ± 1.5	0.001
Max. inclination (%)	5.2 ± 4.8	6.1 ± 4.7	0.049
Distance (m)	1091.2 ± 384.1	1262.9 ± 307.1	0.001
Time (min)	8.6 ± 1.5	11.5 ± 2.1	0.001
**Cardiovascular**			
Resting HR (bpm)	71.47 ± 11.3	79.0 ± 12.0	0.042
Peak HR (bpm)	175.9 ± 23.0	185.8 ± 19.7	0.031
Resting SBP (mmHg)	106.8 ± 21.4	106.2 ± 19.0	NS
Delta SBP (mmHg)	36.1 ± 1.1	39.2 ± 0.9	0.001
_PEAK_ O_2 _Pulse_ _mL.beat^-1^	10.4 ± 5.5	13.5 ± 3.6	0.001
_AT_ O_2 _Pulse mL.beat^-1^	8.3 ± 5.1	12.5 ±3.2	0.001
OUES	1693.5 ± 761.9	1876.6 ± 564.5	0.0001
OUES/kg	34.1 ± 11.1	46.1 ± 9.2	0.0001
Circul. pow. (mmHg/mL/kg)	1924.0 ± 550	3937.5 ± 1220	0.0001
**Metabolic**			
_PEAK_ VO_2 _mL.min^-1^	1021 ± 474.2	1637.40 ± 834.0	0.0001
_PEAK _VO_2 _mL.kg.min^-1^	31.5 ± 7.2	42.3 ± 7.0	0.0001
VO_2 AT _mL.min^-1^	19.5 ± 4.5	25.9 ± 5.3	0.0001
VO_2 AT _mL.min^-1^	643.4 ± 301.8	1004.2 ± 567.5	0.0001
R (VCO_2_/VO_2_)	1.02 ± 0.1	1.04 ± 0.1	NS
PETCO_2_ mmHg	30.83 ± 4.5	34.2 ± 4.0	0.0001
**Ventilatory and gas exchanges**			
Peak VE L.min^-1^	50.4± 22.0	55.2 ± 22.2	0.38
RR (rpm)	61.0 ± 15.2	58.6 ± 10.9	NS
PETCO_2 _mmHg	32.83 ± 3.90	34.41 ± 3.29	0.0005
VE/VCO_2_ slope	41.2 ± 6.40	35.5 ± 4.3	0.0001
O_2 _Sat (%)	90.9 ± 8.2	97.6 ± 1.2	0.0001

AT: anaerobic threshold; HR: heart rate; peak HR: maximal HR reached; delta SBP: difference between peak and resting systolic blood pressure; OUES: oxygen uptake efficiency slope; Circul. pow.: circulatory power; _PEAK_VO_2_: oxygen consumption at peak exertion; VO_2 AT_: oxygen consumption at anaerobic threshold; VE: pulmonary ventilation; RR: respiratory rate; O_2_ Sat (%): oxyhemoglobin saturation (modified from reference 86); NS: non-significant.

Kempny et al.^87^ have reported the reference values of the major ergospirometric variables of adults with congenital heart disease, and have correlated their data with those in the literature to guide the recreational, sports and professional activities of those individuals.

Thus, the association of cardiovascular variables, such as O_2_ pulse and peak VO_2_, and ventilatory variables (VE/VCO_2_ slope) provides more comprehensive and objective data about the true functional capacity of children and adolescents with congenital heart disease. We provide major examples: after late correction of Fallot tetratogy, evolution with pulmonary insufficiency and possible right ventricular dilation and dysfunction can indicate exchange or, currently, implantation of new prostheses, such as Melody's*.*^88,89^ CPET can indicate the best time for intervention, when the morphology of the O_2_ pulse curve shows a depression or early plateau, in addition to ventilatory inefficiency characterized by high VE/VCO_2_ slope values. After late correction of transposition of the great arteries according to Mustard's or Senning's technique, an older method, many children show worsening of their metabolic efficiency (more reduced peak VO_2_ and excessive ventilation - greater VE/VCO_2_ slope), which does not occur when submitted to Jatene's surgery, considered the ideal technique. Additionally, CPET allows the analysis of gas exchange in other more complex congenital heart diseases with pulmonary hypertension, such as Eisenmenger's syndrome.^88,89^

CPET has been a valuable complementary resource in the follow-up of patients with congenital heart diseases to both assess the exercise capacity and indicate the ideal time for new therapeutic approaches, providing objective, diagnostic and prognostic information on the patient's true cardiopulmonary functional status.

## References

[1] Sociedade Brasileira de Cardiologia (2010). III Guidelines of Sociedade Brasileira de Cardiologia on the
exercise test. Arq Bras Cardiol.

[2] Herdy AH, Uhnlerdorf D (2011). Reference values for cardiopulmonary exercise testing for
sedentary and active men and women. Arq Bras Cardiol.

[3] Guazzi M, Adams V, Conraads V, Halle M, Mezzani A, Vanhees L, European Association for Cardiovascular Prevention &
Rehabilitation; American Heart Association. EACPR/AHA Scientific
Statement (2012). Clinical recommendations for cardiopulmonary exercise testing
data assessment in specific patient populations. Circulation.

[4] Piepoli MF, Corra U, Agostoni PG, Belardinelli R, Cohen-Solal A, Hambrecht R, Task Force of the Italian Working Group on Cardiac Rehabilitation
Prevention, Working Group on Cardiac Rehabilitation and Exercise Physiology of
the European Society of Cardiology (2006). Statement on cardiopulmonary exercise testing in chronic heart
failure due to left ventricular dysfunction: recommendations for performance
and interpretation. Part I: definition of cardiopulmonary exercise testing
parameters for appropriate use in chronic heart failure. Eur J Cardiovasc Prev Rehabil.

[5] Task Force of the Italian Working Group on Cardiac Rehabilitation
and Prevention (Gruppo Italiano di Cardiologia Riabilitativa e
Prevenzione, GICR), Working Group on Cardiac Rehabilitation and Exercise Physiology of
the European Society of Cardiology (2006). Statement on cardiopulmonary exercise testing in chronic heart
failure due to left ventricular dysfunction: recommendations for performance
and interpretation Part III: Interpretation of cardiopulmonary exercise
testing in chronic heart failure and future applications. Eur J Cardiovasc Prev Rehabil.

[6] Wasserman K, Whipp BJ (1975). Exercise physiology in health and disease. Am Rev Resp Dis.

[7] Almeida AE, Stefani Cde M, Nascimento JA, Almeida NM, Santos Ada C, Ribeiro JP (2014). An equation for the prediction of oxygen consumption in a
Brazilian population. Arq Bras Cardiol.

[8] Sorajja P, Allison T, Hayes C, Nishimura RA, Lam CS, Ommen SR (2012). Prognostic utility of metabolic exercise testing in minimally
symptomatic patients with obstructive hypertrophic
cardiomyopathy. Am J Cardiol.

[9] Corra U, Giordano A, Bosimini E, Mezzani A, Piepoli M, Coats AJ (2002). Oscillatory ventilation during exercise in patients with chronic
heart failure: clinical correlates and prognostic
implications. Chest.

[10] Taivassalo T, Dysgaard Jensen T, Kennaway N, DiMauro S, Vissing J, Haller RG (2003). The spectrum of exercise tolerance in mitochondrial myopathies: a
study of 40 patients. Brain.

[11] Arena R, Myers J, Guazzi M (2008). The clinical and research applications of aerobic capacity and
ventilatory efficiency in heart failure: an evidence-based
review. Heart Fail Rev.

[12] Torchio R, Guglielmo M, Giardino R, Ardissone F, Ciacco C, Gulotta C (2010). Exercise ventilatory inefficiency and mortality in patients with
chronic obstructive pulmonary disease undergoing surgery for non-small-cell
lung cancer. Eur J Cardiothorac Surg.

[13] Ramos PS, Ricardo DR, Araújo CG (2012). Cardiorespiratory optimal point: a submaximal variable of the
cardiopulmonary exercise testing. Arq Bras Cardiol.

[14] Davies LC, Wensel R, Georgiadou P, Cicoira M, Coats AJ, Piepoli MF (2006). Enhanced prognostic value from cardiopulmonary exercise testing
in chronic heart failure by non-linear analysis: oxygen uptake efficiency
slope. Eur Heart J.

[15] Scrutinio D, Passantino A, Lagioia R, Napoli F, Ricci A, Rizzon P (1998). Percent achieved of predicted peak exercise oxygen uptake and
kinetics of recovery of oxygen uptake after exercise for risk stratification
in chronic heart failure. Int J Cardiol.

[16] Forman DE, Guazzi M, Myers J, Chase P, Bensimhon D, Cahalin LP (2012). Ventilatory power: a novel index that enhances prognostic
assessment of patients with heart failure. Circ Heart Fail.

[17] Araújo CG (2012). Devemos substituir o teste ergométrico convencional pelo
teste cardiopulmonar de exercício. Rev DERC.

[18] Araújo CG, Herdy AH, Stein R (2013). Maximum oxygen consumption measurement: valuable biological
marker in health and in sickness. Arq Bras Cardiol.

[19] Jones NL, Ehrsam RE (1982). The anaerobic threshold. Exerc Sport Sci Rev.

[20] Herdy AH, López-Jimenez F, Terzic CP, Milani M, Stein R, Carvalho T (2014). South American guidelines for cardiovascular disease prevention
and rehabilitation. Arq Bras Cardiol.

[21] Araújo CG (1998). Importância da ergoespirometria na
prescrição de exercício ao cardiopata. Rev SOCERJ.

[22] de Araújo CG (1983). Cardiorespiratory responses to prolonged submaximal
exercise. Arq Bras Cardiol.

[23] Franklin BA, Gordon S, Timmis GC (1996). Diurnal variation of ischemic response to exercise in patients
receiving a once-daily dose of beta-blockers. Implications for exercise
testing and prescription of exercise and training heart
rates. Chest.

[24] Laplaud D, Guinot M, Favre-Juvin A, Flore P (2006). Maximal lactate steady state determination with a single
incremental test exercise. Eur J Appl Physiol.

[25] Bocchi EA, Marcondes-Braga FG, Bacal F, Ferraz AS, Albuquerque D, Rodrigues Dde A (2012). Updating of the Brazilian guideline for chronic heart failure -
2012. Arq Bras Cardiol.

[26] Yancy CW, Jessup M, Bozkurt B, Butler J, Casey DE, Drazner MH, American College of Cardiology FoundationAmerican Heart Association
Task Force on Practice Guidelines (2013). ACCF/AHA guideline for the management of heart failure: a report
of the American College of Cardiology Foundation/American Heart Association
Task Force on Practice Guidelines. J Am Coll Cardiol.

[27] Bacal F, Souza-Neto JD, Fiorelli AI, Mejia J, Marcondes-Braga FG, Mangini S (2009). II Diretriz Brasileira de Transplante
Cardíaco. Arq Bras Cardiol.

[28] Weber KT, Kinasewitz GT, Janicki JS, Fishman AP (1982). Oxygen utilization and ventilation during exercise in patients
with chronic cardiac failure. Circulation.

[29] Chua TP, Ponikowski P, Harrington D, Anker SD, Webb-Peploe K, Clark AL (1997). Clinical correlates and prognostic significance of the
ventilatory response to exercise in chronic heart failure. J Am Coll Cardiol.

[30] Arena R, Myers J, Aslam SS, Varughese EB, Peberdy MA (2004). Peak VO2 and VE/VCO_2_ slope in patients with heart
failure: a prognostic comparison. Am Heart J.

[31] Francis DP, Shamim W, Davies LC, Piepoli MF, Ponikowski P, Anker SD (2000). Cardiopulmonary exercise testing for prognosis in chronic heart
failure: continuous and independent prognostic value from VE/VCO_2_
slope and peak VO2. Eur Heart J.

[32] Corra U, Mezzani A, Bosimini E, Scapellato F, Imparato A, Giannuzzi P (2002). Ventilatory response to exercise improves risk stratification in
patients with chronic heart failure and intermediate functional
capacity. Am Heart J.

[33] Ritt LE, Carvalho AC, Feitosa GS, Pinho-Filho JA, Andrade MV, Feitosa-Filho GS (2013). Cardiopulmonary exercise and 6-min walk tests as predictors of
quality of life and long-term mortality among patients with heart failure
due to Chagas disease. Int J Cardiol.

[34] Arena R, Myers J, Abella J, Peberdy MA, Bensimhon D, Chase P (2007). Development of a ventilatory classification system in patients
with heart failure. Circulation.

[35] Ferreira AM, Tabet JY, Frankenstein L, Metra M, Mendes M, Zugck C (2010). Ventilatory efficiency and the selection of patients for heart
transplantation. Circ Heart Fail.

[36] Baba R, Nagashima M, Goto M, Nagano Y, Yokota M, Tauchi N (1996). Oxygen intake efficiency slope: a new index of cardiorespiratory
functional reserve derived from the relationship between oxygen consumption
and minute ventilation during incremental exercise. Nagoya J Med Sci.

[37] Hollenberg M, Tager IB (2000). Oxygen uptake efficiency slope: an index of exercise performance
and cardiopulmonary reserve requiring only submaximal
exercise. J Am Coll Cardiol.

[38] de Groote P, Millaire A, Decoulx E, Nugue O, Guimier P, Ducloux (1996). Kinetics of oxygen consumption during and after exercise in
patients with dilated cardiomyopathy. New markers of exercise intolerance
with clinical implications. J Am Coll Cardiol.

[39] Reddy HK, Weber KT, Janicki JS, McElroy PA (1988). Hemodynamic, ventilatory and metabolic effects of light isometric
exercise in patients with chronic heart failure. J Am Coll Cardiol.

[40] Nishime EO, Cole CR, Blackstone EH, Pashkow FJ, Lauer MS (2000). Heart rate recovery and treadmill exercise score as predictors of
mortality in patients referred for exercise ECG. JAMA.

[41] Arena R, Myers J, Abella J, Peberdy MA, Bensimhon D, Chase P (2010). The prognostic value of the heart rate response during exercise
and recovery in patients with heart failure: influence of
beta-blockade. Int J Cardiol.

[42] Ritt LE, Oliveira RB, Myers J, Arena R, Peberdy MA, Bensimhon D (2012). Patients with heart failure in the "intermediate range" of peak
oxygen uptake: additive value of heart rate recovery and the minute
ventilation/carbon dioxide output slope in predicting
mortality. J Cardiopulm Rehabil Prev.

[43] Leite JJ, Mansur AJ, de Freitas HF, Chizola PR, Bocchi EA, Terra-Filho M (2003). Periodic breathing during incremental exercise predicts mortality
in patients with chronic heart failure evaluated for cardiac
transplantation. J Am Coll Cardiol.

[44] Murphy RM, Shah RV, Malhotra R, Pappagianopoulos PP, Hough SS, Systrom DM (2011). Exercise oscillatory ventilation in systolic heart failure: an
indicator of impaired hemodynamic response to exercise. Circulation.

[45] Sun XG, Hansen JE, Beshai JF, Wasserman K (2010). Oscillatory breathing and exercise gas exchange abnormalities
prognosticate early mortality and morbidity in heart failure. J Am Coll Cardiol.

[46] Arena R, Guazzi M, Myers J (2007). Prognostic value of end-tidal carbon dioxide during exercise
testing in heart failure. Int J Cardiol.

[47] Oliveira RB, Myers J, Araújo CG, Arena R, Mandic S, Bensimhon D (2009). Does peak oxygen pulse complement peak oxygen uptake in risk
stratifying patients with heart failure. Am J Cardiol.

[48] Levy WC, Arena R, Wagoner LE, Dardas T, Abraham WT (2012). Prognostic impact of the addition of ventilatory efficiency to
the Seattle Heart Failure Model in patients with heart
failure. J Card Fail.

[49] Cahalin LP, Chase P, Arena R, Myers J, Bensimhon D, Peberdy MA (2013). A meta-analysis of the prognostic significance of cardiopulmonary
exercise testing in patients with heart failure. Heart Fail Rev.

[50] Myers J, Oliveira R, Dewey F, Arena R, Guazzi M, Chase P (2013). Validation of a cardiopulmonary exercise test score in heart
failure. Circ Heart Fail.

[51] Ritt LE, Myers J, Stein R, Arena R, Guazzi M, Chase P (2015). Additive prognostic value of a cardiopulmonary exercise test
score in patients with heart failure and intermediate risk. Int J Cardiol.

[52] Belardinelli R, Lacalaprice F, Carle F, Minnucci A, Cianci G, Perna G (2003). Exercise-induced myocardial ischaemia detected by cardiopulmonar
exercise testing. Eur Heart J.

[53] Munhoz EC, Hollanda R, Vargas JP, Silveira CW, Lemos AL, Hollanda RM (2007). Flattening of oxygen pulse during exercise may detect extensive
myocardial ischemia. Med Sci Sports Exerc.

[54] Lavie CJ, Milani RV, Mehra MR (2004). Peak exercise oxygen pulse and prognosis in chronic heart
failure. Am J Cardiol.

[55] Belardinelli R, Lacalaprice F, Tiano L, Muçai A, Perna GP (2014). Cardiopulmonary exercise testing is more accurate than ECG-stress
testing in diagnosing myocardial ischemia in subjects with chest
pain. Int J Cardiol.

[56] Pinkstaff S, Peberdy MA, Kontos MC, Fabiato A, Finucane S, Arena R (2010). Usefulness of decrease in oxygen uptake efficiency slope to
identify myocardial perfusion defects in men undergoing myocardial ischemic
evaluation. Am J Cardiol.

[57] Klainman E, Fink G, Lebzelter J, Krelbaumm T, Kramer MR (2008). The relationship between left ventricular function assessed by
multigated radionuclide test and cardiopulmonary exercise test in patients
with ischemic heart disease. Chest.

[58] Inbar O, Yamic C, Bar-On I, Nice S, David D (2008). Effects of percutaneous transluminal coronary angioplasty on
cardiopulmonary responses during exercise. J Sports Med Phys Fitness.

[59] Morgan WC, Hodge HL (1998). Diagnostic evaluation of dyspnea. Am Fam Physician.

[60] Wahls SA (2012). Causes and evaluation of chronic dyspnea. Am Fam Physician.

[61] Messner-Pellence P, Ximenes C, Brasileiro CF, Mercier J, Grolleau R, Prefaut CG (1994). Cardiopulmonary exercise testing: determinants of dyspnea due to
cardiac or pulmonary limitation. Chest.

[62] Palange P, Ward SA, Calsen KH, Casaburi R, Gallagher CG, Grosselink R, ERS Task Force (2007). Recommendations on the use of exercise testing in clinical
practice. Eur Respir J.

[63] Beaver WL, Wasserman K, Whipp BJ (1973). On-line computer analysis and breath-by-breath graphical display
of exercise function tests. J Appl Physiol.

[64] Arena R, Sietsema KE (2011). Cardiopulmonary exercise testing in the clinical evaluation of
patients with heart and lung disease. Circulation.

[65] Arena R, Myers J, Guazzi M (2011). Cardiopulmonary exercise testing is a core assessment for
patients with heart failure. Congest Heart Fail.

[66] McNicholl DM, Megarry J, McGarvey LP, Riley MS, Heaney LG (2011). The utility of cardiopulmonary exercise testing in difficult
asthma. Chest.

[67] Pasnick SD, Carlos 3rd WG, Arunachalam A, Celestin FM, Parsons JP, Hallstrand TS, American Thoracic Society Implementation Task Force (2014). Exercise - induced bronchoconstriction. Ann Am Thorac Soc.

[68] Sun XG, Hansen JE, Oudiz RJ, Wasserman K (2001). Exercise pathophysiology in patients with primary pulmonary
hypertension. Circulation.

[69] Galiè N, Hoeper MM, Humbert M, Torbicki A, Vachiery JL, Barbera JA, Task Force for Diagnosis, Treatment of Pulmonary Hypertension of European Society of
Cardiology, European Respiratory Society, International Society of Heart and Lung Transplantation (2009). Guidelines for the diagnosis and treatment of pulmonary
hypertension. Eur Respir J.

[70] Galie N, Hoeper MM, Humbert M, Torbicki A, Vachiery JL, Barbera JA (2009). Guidelines for the diagnosis and treatment of pulmonary
hypertension: the Task Force for the Diagnosis and Treatment of Pulmonary
Hypertension of the European Society of Cardiology (ESC) and the European
Respiratory Society (ERS), endorsed by the International Society of Heart
and Lung Transplantation (ISHLT). Eur Heart J.

[71] Palange P, Ward SA, Carlsen KH, Casaburi R, Gallagher CG, ERST Task Force (2007). Recommendations on the use of exercise testing in clinical
practice. Eur Respir J.

[72] Kovacs G, Berghold A, Scheidl S, Olschewski H (2009). Pulmonary arterial pressure during rest and exercise in healthy
subjects: a systematic review. Eur Respir J.

[73] Deboeck G, Niset G, Lamotte M, Vachiery JL, Naeije R (2004). Exercise testing in pulmonary arterial hypertension and in
chronic heart failure. Eur Respir J.

[74] Yasunobu Y, Oudiz RJ, Sun XG, Hansen JE, Wasserman K (2005). End-tidal PCO2 abnormality and exercise limitation in patients
with primary pulmonary hypertension. Chest.

[75] Wensel R, Opitz CF, Anker SD, Winkler J, Hoffken G, Kleber FX (2002). Assessment of survival in patients with primary pulmonary
hypertension: importance of cardiopulmonary exercise testing. Circulation.

[76] Zhai Z, Murphy K, Tighe H, Wang C, Wilkins MR, Gibbs JS (2011). Differences in ventilatory inefficiency between pulmonary
arterial hypertension and chronic thromboembolic pulmonary
hypertension. Chest.

[77] Oudiz RJ, Midde R, Hovenesyan A, Sun XG, Roveran G, Hansen JE (2010). Usefulness of right-to-left shunting and poor exercise gas
exchange for predicting prognosis in patients with pulmonary arterial
hypertension. Am J Cardiol.

[78] Johnson J, Yetman AT (2012). Cardiopulmonary exercise testing in adults with congenital heart
disease. Progress in Pediatric Cardiology.

[79] Takken T, Blank AC, Hulzebos BH, van Brussel M, Groen WG, Helders PJ (2009). Cardiopulmonary exercise testing in congenital heart disease:
equipment and test protocols. Neth Heart J.

[80] Cooper DM, Kaplan MR, Baumgarten L, Weiler-Ravell D, Whipp BJ, Wasserman K (1987). Coupling of ventilation and CO2 production during exercise in
children. Pediatr Res.

[81] Prado DM, Dias RG, Trombetta IC (2006). Cardiovascular, ventilatory and metabolic parameters during
exercise: differences between children and adults. Arq Bras Cardiol.

[82] Kuno S, Takahashi H, Fujimoto K, Akima H, Miyamaru M, Nemoto I (1995). Muscle metabolism during exercise using phosphorus-31 nuclear
magnetic resonance spectroscopy in adolescents. Eur J Appl Physiol.

[83] Eriksson BO, Karlsson J, Saltin B (1971). Muscle metabolites during exercise in pubertal
boys. Acta Pediatr Scand.

[84] Eriksson BO, Gollnick PD, Saltin B (1973). Muscle metabolism and enzyme activities after training in boys
11- 13 years old. Acta Physiol Scand.

[85] Boisseau N, Delamarche P (2000). Metabolic and hormonal responses to exercise in children and
adolescents. Sports Med.

[86] Aguiar F° GB, Hossri CA, Dias LT, Meneghelo RS Avaliação das variáveis cardiometabólicas em
crianças e adolescentes no pós-operatório tardio de
correção de cardiopatias congênitas.

[87] Kempny A, Dimopoulos K, Uebing A, Moceri P, Swan L, Gatzoulis MA (2012). Reference values for exercise limitations among adults with
congenital heart disease. Relation to activities of daily life-single centre
experience and review of published data. Eur Heart J.

[88] Gatzoulis MA, Clark AL, Cullen S, Claus GH, Redington AN (1995). Right ventricular diastolic function 15 to 35 years after repair
of tetralogy of Fallot. Circulation.

[89] Baumgartner H, Bonhoeffer P, De Groot NM, de Haan F, Deanfield JE, Galie N (2010). Task Force on the Management of Grown-up Congenital Heart Disease
of the European Society of Cardiology (ESC). ESC Guidelines for the
management of grown-up congenital heart disease (new version
2010). Eur Heart J.

